# The Common Task

**Published:** 1907-06-08

**Authors:** 


### THE COMMON TASK.

Correspondence and Queries for this section should be sent to the Editor of The Hospital, 28 Southampton Street, Strand, London, and marked "Nursing Administration."

### Clean Milk.

The plan adopted by the Cardiff Infirmary to ensure a pure milk supply is worthy of imitation. The form of tender lays down as a condition that the cows shall be examined and reported on by a veterinary surgeon selected by the Infirmary, and the cows thus passed as free from disease shall be the only ones whose milk shall be sent to the Infirmary. The dealer shall undertake to wash the udders of the cows with soap and water just before milking the animals, and shall further undertake to see that those who milk the cows shall wash their hands immediately before milking. The cows shall be milked in a place free from manure and dirt. The milk is to be strained and cooled to at least 50° F. immediately after milking, and then placed in churns belonging to the Infirmary, which shall be at once locked down, and the milk conveyed to the Infirmary without delay. All this is excellent, and the right of inspection being also secured, the Cardiff Infirmary must be able to feel tolerably secure about the cleanliness of their milk, due precautions being taken also to ensure that its constituent qualities are satisfactory. It would be interesting to know how long milk supplied under these favourable conditions will keep sweet in a cool dairy.

### Entrance Fees for Probationers.

Small premiums of a guinea or so for probationers are found, it appears, very useful in freeing the hospital from enterprising ladies who are seized with a desire to see what nursing is like, but have no real intention of ever remaining to complete their course. Some provincial hospitals are much plagued with these restless persons, and even a small fee does much to prevent the hospital from being used as a convenient spot in which to get a change of ideas, and a relief for a few weeks from the monotony of home life.

### The Paying Ward as a Factor in Training.

In hospitals of such size that probationers cannot possibly expect to get experience in all the wards in the course of their three years' training, the paying wards are a valuable part of the building. It is not merely that the conditions approximate more nearly to those obtaining in private nursing than can be the case in the general wards, but the variety of cases found side by side, unsorted, if the expression may be permitted, gives the probationer a more general idea of both surgical and medical work than is possible in wards devoted entirely to one particular section of the body. It may almost be asserted that the probationer who is fortunate enough to spend her third year at Guy's in the Bright Ward gets a complete education in nursing irrespective of her previous experience. For here, and indeed in most paying wards in modern hospitals, there is a private theatre, and thus the privilege of experience in a branch of work which cannot possibly in a large hospital be accorded to all, is added to her general duties. The difference in nursing in the pay wards as contrasted with that in the ordinary part of the hospital resolves itself mainly into a difference in the service of meals, the private patients getting trays where the ordinary patients get plates. But the divisions of the ward into cubicles, which prevents the nurse from keeping it all under observation at the same time, renders more hands necessary, and keeps the staff perpetually busy. This is hardly more of an addition to the responsibilities of the ward sister, however, than the balcony system, under which a good section of the beds are removed from immediate observation. If for no other reason than for helping to rid the nursing staff of routine, the provision of paying wards in general hospitals ought to be welcome.

### Instruction in Institution Management.

A complaint comes from America that superintendents of nurses have no facilities afforded them of acquiring knowledge of their administrative duties before taking up responsible posts. It is idle, however, to advocate the establishment of schools for superintendents. There can be no way of learning the secrets of a good administrator except by doing the work, and although much may be learned by serving as assistants the real difficulties of the head of a department can never be gauged except by one who holds a similar post.

